# Enzalutamide Enhances PSMA Expression of PSMA-Low Prostate Cancer

**DOI:** 10.3390/ijms22147431

**Published:** 2021-07-11

**Authors:** Magdalena Staniszewska, Pedro Fragoso Costa, Matthias Eiber, Jasmin M. Klose, Jasmin Wosniack, Henning Reis, Tibor Szarvas, Boris Hadaschik, Katharina Lückerath, Ken Herrmann, Wolfgang P. Fendler, Janette Iking

**Affiliations:** 1 Department of Nuclear Medicine, University of Duisburg-Essen and German Cancer Consortium (DKTK)-University Hospital Essen, Hufelandstraße 55, D-45147 Essen, Germany; Magdalena.Staniszewska@uk-essen.de (M.S.); Pedro.FragosoCosta@uk-essen.de (P.F.C.); Jasmin.Klose@uk-essen.de (J.M.K.); Jasmin.Wosniack@uk-essen.de (J.W.); Katharina.Lueckerath@uk-essen.de (K.L.); Ken.Herrmann@uk-essen.de (K.H.); 2 Department of Nuclear Medicine, Klinikum Rechts der Isar, Technical University of Munich, Ismaninger Straße 22, D-81675 Munich, Germany; matthias.eiber@tum.de; 3 Institute of Pathology, University of Duisburg-Essen, University Hospital Essen, Hufelandstraße 55, D-45147 Essen, Germany; Henning.Reis@uk-essen.de; 4 Department of Urology, University of Duisburg-Essen and German Cancer Consortium (DKTK)-University Hospital Essen, Hufelandstraße 55, D-45147 Essen, Germany; Tibor.Szarvas@uk-essen.de (T.S.); Boris.Hadaschik@uk-essen.de (B.H.); 5 Department of Urology, Semmelweis University, Ulloi ut 78/b, 1082 Budapest, Hungary; 6 Department of Molecular and Medical Pharmacology, David Geffen School of Medicine, University of California Los Angeles, 650 Charles E Young Drive S, Los Angeles, CA 90095, USA

**Keywords:** PSMA, prostate cancer, enzalutamide, androgen receptor blockade, PET, CT, radioligand therapy, 22Rv1, C4-2, LNCaP

## Abstract

Prostate-specific membrane antigen (PSMA)-directed radioligand therapy (RLT) prolongs overall survival in men with metastatic castration-resistant prostate cancer (mCRPC). However, men with low PSMA expression are excluded from RLT. We explored the effect of androgen receptor blockade with enzalutamide on PSMA expression. Assessment of PSMA and androgen receptor (AR) expression on the human PC cell lines 22Rv1, C4-2, and LNCaP by immunohistochemistry and flow cytometry revealed low (22Rv1) and high (C4-2 and LNCaP) PSMA expression, and high, comparable AR positivity. Treatment with enzalutamide increased PSMA levels in 22Rv1, C4-2, and LNCaP (2.2/2.3/2.6-fold, *p* = 0.0005/0.03/0.046) after one week compared to DMSO-treated controls as assessed by flow cytometry. NOD/Scid mice bearing 22Rv1 tumors were treated with enzalutamide for two weeks. Positron emission tomography/computed tomography (PET/CT) demonstrated higher tumor uptake of ^68^Ga-PSMA after enzalutamide treatment (*p* = 0.004). Similarly, a clinical case with low baseline PSMA avidity demonstrated increased uptake of ^68^Ga-PSMA after enzalutamide on PET/CT and post-therapeutic ^177^Lu-PSMA scintigraphy in a patient with mCRPC. Enzalutamide induced PSMA expression in the 22Rv1 xenograft model and in an mCRPC patient, both with low baseline tumoral PSMA levels. Therefore, enzalutamide pre-treatment might render patients with low PSMA expression eligible for ^177^Lu-PSMA RLT.

## 1. Introduction

Androgen deprivation therapy (ADT) and androgen receptor blockade (ARB) are important components of prostate cancer (PC) management [1]. However, over time metastatic PC becomes resistant to androgen receptor-targeted therapy and progresses within 18 to 36 months towards castration-resistant PC (CRPC), the lethal late stage of the disease [2]. Therapeutic options for men with CRPC have improved in recent years. Second-generation ARB, for example, improves patient survival and overall quality of life. Enzalutamide increases median radiographic progression-free (rPFS) survival by 14.6 months, and median overall survival (OS) before chemotherapy, despite cross-over, by 4 months when compared to placebo [3]. A more recent study confirms OS benefit from enzalutamide in hormone-sensitive PC [4]. Radioligand therapy (RLT) targeting the prostate-specific membrane antigen (PSMA) is a new therapeutic option for CRPC patients [5,6,7]. In the phase 3 VISION trial, PSMA-RLT combined with prior or concurrent ARB was reported to improve both rPFS and OS (NCT03511664) [8,9,10]. Several studies reported that AR suppresses PSMA transcription while ADT/ARB leads to de-repression [11,12,13,14,15,16]. At present, patients with PSMA-low PC are not eligible for RLT and might benefit from PSMA enhancing therapy to enable subsequent RLT. While it has been shown in a model of PSMA-high PC that ARB-induced increases in PSMA expression can lead to higher numbers of DNA double-strand breaks following RLT [16], data on the impact of enzalutamide on PSMA levels in PSMA-low PC are lacking so far. Here, we assess in vitro, in vivo and in an mCRPC clinical case whether enzalutamide treatment can induce PSMA expression in PSMA-low, RLT-ineligible prostate cancer.

## 2. Results

### 2.1. PSMA and AR Expression in Three Different PC Cell Lines

**Figure 1:**
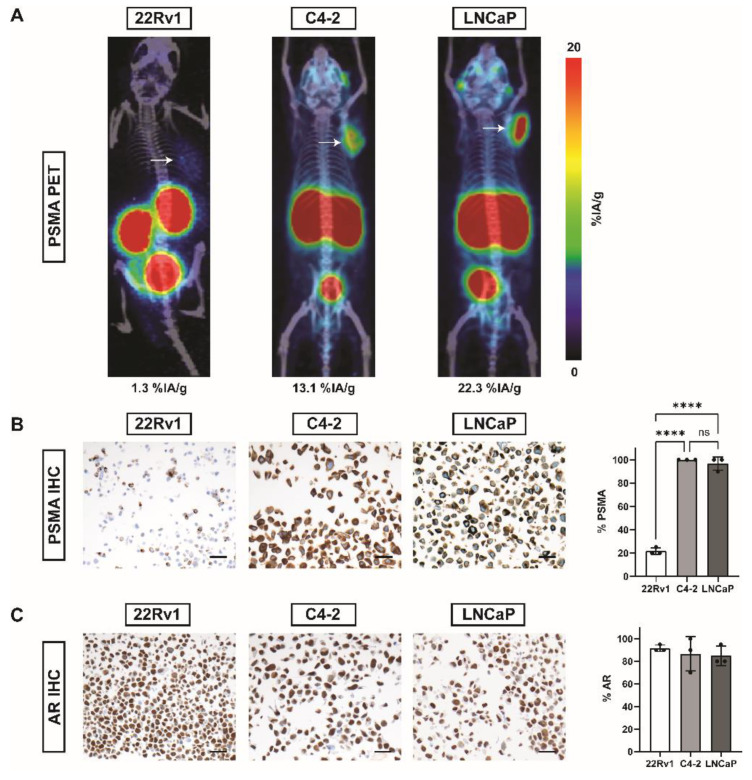
PSMA and AR in 22Rv1, C4-2, and LNCaP prostate cancer cell lines by PET and IHC. (**A**) Exemplary ^68^Ga-PSMA-11-PET/CT maximum intensity projection (MIP) images of NOD/Scid mice bearing tumors (white arrows) of 22Rv1, C4-2, or LNCaP cell lines. Average tumor uptake (%IA/g) per gram tissue is stated below the image. (**B**) Representative images of PSMA IHC and bar plots for % positivity (mean ± SD, *n* = 3). (**C**) Representative images of AR IHC and bar plots for % positivity (mean ± SD, *n* = 3). Scale = 20 µm. Abbreviations: PSMA, prostate-specific membrane antigen; %IA/g, % injected activity per gram tissue; AR, androgen receptor. Statistics: ordinary one-way ANOVA with Sidak’s multiple comparisons test; **** *p* ≤ 0.0001; ns = non significant.

^68^Ga-PSMA tumor uptake assessed by positron emission tomography/computed tomography (PET/CT) was 1.3% injected activity per gram tissue (%IA/g) for 22Rv1 xenografts, 13.1%IA/g for C4-2 and 22.3%IA/g for LNCaP tumors, respectively (Figure 1A). IHC detecting PSMA showed significantly lower expression of PSMA in 22Rv1 (mean ± SD: 21.7% ± 2.9% PSMA^+^ cells; *p* < 0.0001 vs. C4-2 and LNCaP; *n* = 3) cells compared to C4-2 (mean ± SD: 100.0% ± 0% PSMA^+^ cells) and LNCaP (mean ± SD: 96.7% ± 5.8% PSMA^+^ cells) cells (Figure 1B). Androgen receptor (AR) was highly expressed in all three cell lines: 22Rv1 (mean ± SD: 91.7% ± 2.9% AR^+^ cells), C4-2 (mean ± SD: 86.7% ± 15.3% AR^+^ cells) and LNCaP (mean ± SD: 85.0% ± 8.7% AR^+^ cells) (Figure 1C).

### 2.2. Enzalutamide Increases PSMA Expression in Three Different PC Cell Lines

**Figure 2:**
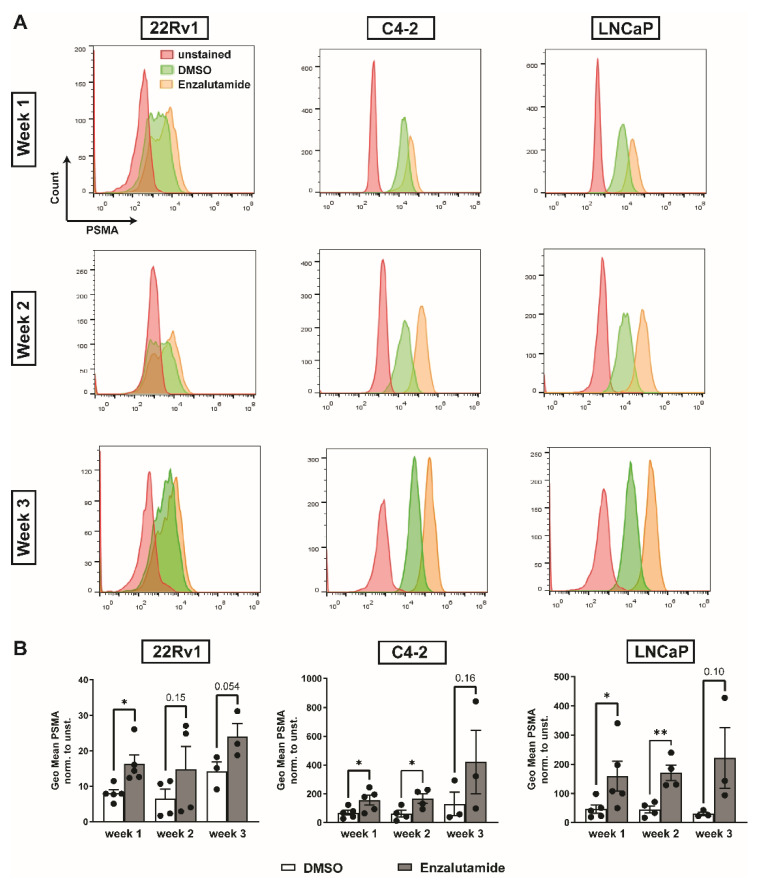
In vitro PSMA expression after enzalutamide treatment over time measured by flow cytometry. (**A**) Representative histograms of unstained and PSMA-stained DMSO-treated or enzalutamide-treated 22Rv1, C4-2 and LNCaP cells. Cells were treated for one, two, or three weeks. (**B**) Enzalutamide treatment significantly increased PSMA levels in 22Rv1, C4-2, and LNCaP cells after one week compared to DMSO-treated controls as assessed by flow cytometry; PSMA levels remained increased in all three cell lines two and three weeks after treatment initiation. Data are shown as geometric mean normalized to unstained control. Statistics: unpaired *t*-test with Welch’s correction; * *p* ≤ 0.05; ** *p* ≤ 0.01.

Enzalutamide significantly increased PSMA expression levels after one week of treatment in all cell lines compared to vehicle-treated controls (DMSO vs. enzalutamide, fold change, mean ± SD: 22Rv1 8.0 ± 2.3 vs. 16.3 ± 5.7, *p* = 0.01; C4-2 67.6 ± 37.3 vs. 155.0 ± 74.6, *p* = 0.03; LNCaP 46.0 ± 31.8 vs. 159.1 ± 114.7, *p* = 0.045, all *n* = 5). PSMA levels remained elevated at two weeks (DMSO vs. enzalutamide, fold change, mean ± SD: 22Rv1 6.5 ± 5.3 vs. 14.75 ± 13.0, *p* = 0.15; C4-2 60.6 ± 47.1 vs. 166.2 ± 65.0, *p* = 0.02; LNCaP 44.7 ± 24.7 vs. 170.8 ± 52.9, *p* = 0.005, all *n* = 4) and three weeks (DMSO vs. enzalutamide, fold change, mean ± SD: 22Rv1 14.2 ± 4.6 vs. 24.0 ± 6.5, *p* = 0.054; C4-2 129.4 ± 142.6 vs. 421.0 ± 382.8, *p* = 0.16; LNCaP 30.0 ± 10.9 vs. 221.5 ± 180.5, *p* = 0.10, all *n* = 3) after treatment start (Figure 2).

### 2.3. PSMA Expression in 22Rv1 Xenografts

**Figure 3:**
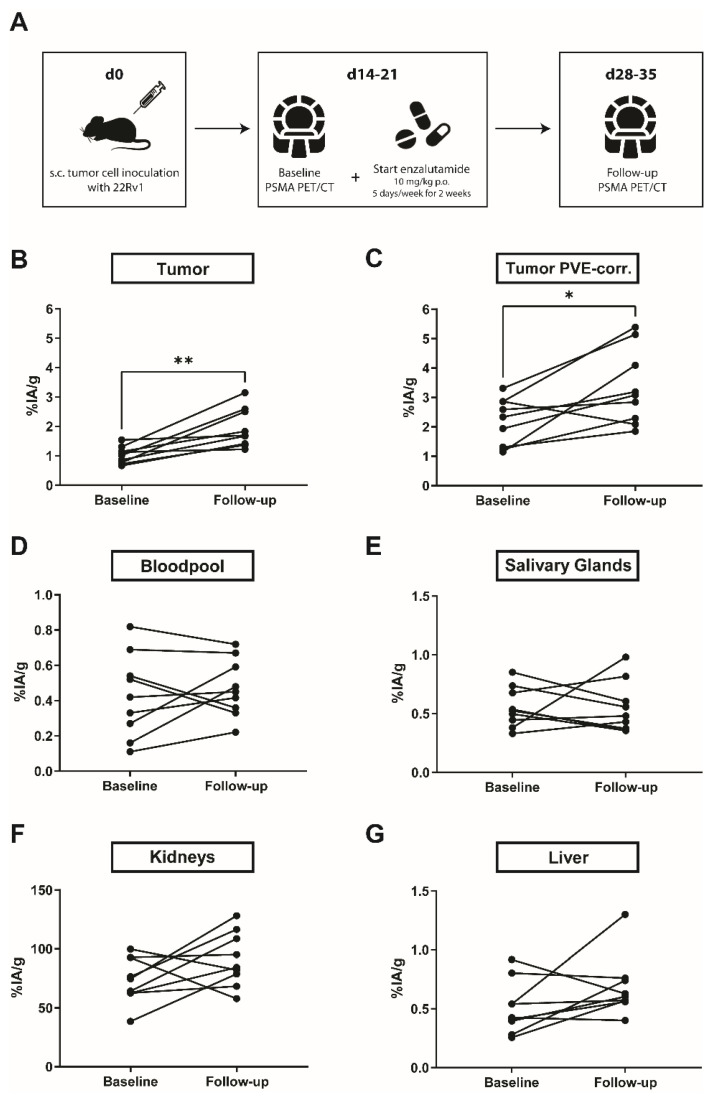
In vivo PSMA levels before and after treatment with enzalutamide by PET/CT. (**A**) 22Rv1 tumor cells were injected into the shoulder region of male, 6–10-week-old NOD/Scid mice. As soon as tumors were palpable (approx. 2–3 weeks after inoculation), baseline ^68^Ga-PSMA-11 PET/CT was performed and enzalutamide treatment (10 mg/kg daily by oral gavage (p.o.), 5 days a week for 2 weeks) was started. After 2 weeks of enzalutamide, follow-up ^68^Ga-PSMA-11 PET/CT was performed. (**B**) ^68^Ga-PSMA uptake per gram tissue in 22Rv1 tumors increased significantly after two weeks of treatment with enzalutamide. PVE-corrected tumor size before and after enzalutamide is shown in (**C**). Measurements of bloodpool (**D**), salivary glands (**E**), kidneys (**F**) and liver (**G**) served as controls and did not show a significant change in ^68^Ga-PSMA uptake. Abbreviations: %IA/g, % injected activity per gram tissue. Statistics: Wilcoxon matched-pairs signed-rank test. * *p* ≤ 0.05, ** *p* ≤ 0.01.

To further test our hypothesis that enzalutamide can increase PSMA levels in PSMA-low PC, we chose the PSMA-low 22Rv1 model for in vivo assessment. The design for the in vivo experiments is outlined in Figure 3A. ^68^Ga-PSMA-11 PET/CT confirmed enhanced PSMA expression after two weeks of enzalutamide treatment (mean ± SD %IA/g: baseline: 1.0 ± 0.3, follow-up: 1.9 ± 0.7; *p* = 0.004; *n* = 9; Figure 3B; Supplementary Figure S1). Measurements of bloodpool (mean ± SD: baseline: 0.4  ±  0.2 %IA/g, follow-up: 0.5  ±  0.2; *p* = 0.65; *n* = 9; Figure 3D), salivary glands (mean ± SD: baseline: 0.6 ± 0.2 %IA/g, follow-up: 0.6  ±  0.2; *p* = 0.49; *n* = 9; Figure 3E), kidneys (mean ± SD: baseline: 73.9  ±  19.3 %IA/g, follow-up: 91.2  ±  23.0; *p* = 0.10; *n* = 9; Figure 3F), and liver (mean ± SD: baseline: 0.5  ±  0.2 %IA/g, follow-up: 0.7  ±  0.3; *p* = 0.16; *n* = 9; Figure 3G) served as controls and did not show a significant increase in ^68^Ga-PSMA-11 uptake. The mean tumor size at the beginning of enzalutamide treatment was 0.14 ± 0.16 cm^3^ and 1.08 ± 0.47 cm^3^ (*n* = 9) at the last day of therapy (Supplementary Figure S2A). Small volume of interests (VOI) measured in PET (such as small tumors) can lead to an over- or underestimation of the signal measured within the VOI. Because of this so-called partial volume effect, it is difficult to determine whether the increased PSMA signal after enzalutamide is due to the treatment or due to tumor growth. Therefore, ^68^Ga-PSMA-11 uptake in tumors was additionally assessed with a partial volume effect correction (PVC) which confirmed a significant increase in PSMA levels after two weeks of enzalutamide treatment (mean ± SD: baseline: 2.2 ± 0.8 %IA/g, follow-up: 3.3 ± 1.3 %IA/g; *p* = 0.02; *n* = 9; Figure 3C; Supplementary Figure S2B,C).

### 2.4. Enzalutamide Increases ^68^Ga-PSMA-11 Uptake in a Patient with mCRPC

**Figure 4:**
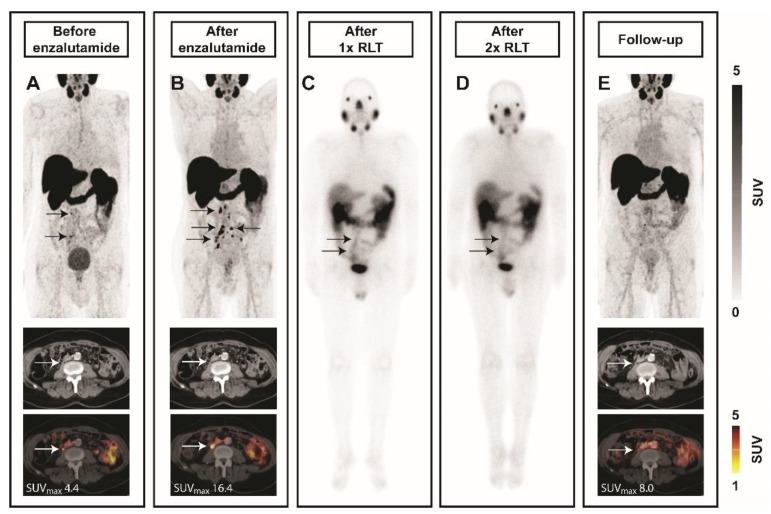
Enhanced PSMA levels under enzalutamide in a patient with low-PSMA nodal metastases. Images from an 81-year-old mCRPC patient are shown. At baseline, a few lymph node metastases with low ^68^Ga-PSMA-11 uptake on PET/CT are present (**A**, arrows). Patient started mCRPC treatment with enzalutamide. A follow-up ^68^Ga-PSMA-11 PET/CT scan after five months showed progressive lymph node disease and increased ^68^Ga-PSMA uptake (**B**, arrows). Subsequently, two cycles of 7.4 GBq ^177^Lu-PSMA radioligand therapy (RLT) were administered with decreasing uptake on the post-therapeutic scintigraphies (**C**–first cycle, **D**–second cycle, arrows). Follow-up ^68^Ga-PSMA-11 PET/CT was consistent with treatment response and demonstrated size decrease of tumor lesions along with reduced PSMA-ligand uptake (**E**, arrows). Abbreviations: SUV, standardized uptake value.

An 81-year-old patient with progressive mCRPC after treatment with abiraterone underwent a baseline ^68^Ga-PSMA-11 PET/CT scan before treatment with enzalutamide. Multifocal pelvic and retroperitoneal lymph node metastases with low ^68^Ga-PSMA-11 uptake on PET/CT were visible (Figure 4A). The patient started treatment with enzalutamide. A follow-up ^68^Ga-PSMA-11 PET/CT scan (Figure 4B) after five months showed progressive lymph node disease and increased ^68^Ga-PSMA-11 uptake sufficient for ^177^Lu-PSMA-617 RLT. Subsequently, the patient received two cycles of 7.4 GBq ^177^Lu-PSMA with confirmed uptake in metastases on the post-therapeutic scintigraphies (Figure 4C,D). Follow-up ^68^Ga-PSMA-11 PET/CT was consistent with treatment response and demonstrated size decrease of involved lymph nodes along with reduced PSMA-ligand uptake (Figure 4E). Prostate-specific antigen (PSA) was non-contributory in this case due to PSA-negative disease.

## 3. Discussion

In this study, we report preclinical data and a clinical case on enhanced PSMA expression after enzalutamide treatment in PSMA-low PC. PSMA, a transmembrane glycoprotein overexpressed on PC cells, has emerged as a novel target for imaging and therapy of PC. High PSMA expression levels are associated with enhanced tumor targeting by RLT and low or heterogeneous PSMA expression represents a resistance mechanism to RLT [9,17,18]. Focal uptake on PSMA PET is a prerequisite for RLT eligibility. Thus, elevating PSMA levels in patients with PSMA-low PC may become an important tool for eligibility and improved efficacy of RLT.

^177^Lu-PSMA RLT is an efficacious therapeutic option for mCRPC. The phase III VISION trial with ^177^Lu-PSMA617 met both primary endpoints; ^177^Lu-PSMA617 significantly improved OS and rPFS in patients with PSMA-positive mCRPC. These encouraging results move PSMA-targeted RLT closer to regulatory approval [10]. However, the VISION trial demonstrated that about 10% of patients will not be eligible for RLT due to insufficient tumoral PSMA expression and more than half of patients will not experience radiographic response after ^177^Lu-PSMA [10]. Thus, there is an urgent need to improve ^177^Lu-PSMA RLT efficacy. It has been reported that PSMA levels can be modulated by androgen receptor blockade in vitro and in vivo [13,15,16,19,20]. However, most studies were performed in models with high basal PSMA expression levels and data on PSMA-low PC is still lacking.

We investigated the effect of ARB on PSMA expression over a 3-week period in PSMA-low human prostate cancer cell line 22Rv1 and PSMA-high human prostate cancer cell lines C4-2 and LNCaP. Our results demonstrate that enzalutamide increases PSMA expression significantly in all three cell lines already after one week of treatment. 22Rv1 expresses low levels of PSMA which was confirmed by immunohistochemistry and flow cytometry and therefore closely represents challenging patient cohorts that will potentially not be eligible for or respond to RLT, as demonstrated in a recent multi-center analysis of clinical predictors of non-response [9].

This finding provided the rationale for investigating the effect of ARB with enzalutamide on PSMA expression in vivo using PSMA-low 22Rv1 tumors. We demonstrated that ^68^Ga-PSMA-11 uptake was significantly increased after two weeks of enzalutamide treatment in the 22Rv1 xenograft model. It was previously shown that androgens inhibit PSMA expression [12,21], which might explain the enhanced PSMA expression after ARB. To clarify the exact mechanism of this phenomenon, more studies are necessary; however, it has been shown that two regulatory elements control PSMA expression: a PSMA promoter and a PSMA enhancer, located within the third intron of PSMA [22]. Androgens form a complex with AR which subsequently translocates to the nucleus and binds to the PSMA enhancer region of the PSMA gene leading to its inactivation. This complex might be responsible for androgen-mediated downregulation of PSMA gene transcription. On the contrary, ADT/ARB competitively inhibits androgen binding to AR and the PSMA enhancer region of PSMA gene is then activated [23,24]. Hope et al., Lückerath et al. and Evans et al. reported enhanced PSMA expression in PSMA-high LNCaP and C4-2 xenografts upon ARB and androgen deprivation, respectively [11,15,16]. Lückerath et al. demonstrated that pre-treatment with enzalutamide before RLT led to more substantial DNA damage (phospho-γH2A.X) compared to RLT monotherapy but did not result in additional tumor growth retardation. A possible explanation might center around the high basal PSMA levels in C4-2 that render C4-2 tumors sensitive to RLT; ARB-induced elevation of PSMA might not translate in further improved RLT efficacy. In agreement with that notion, Current et al. [17] reported that enhanced PSMA levels translated into improved RLT efficacy in RM1 tumors with substantially lower basal PSMA expression compared to C4-2; this finding might suggest that the effect of elevating PSMA levels might be more striking in PSMA-low tumors. Additionally, Roy et al. [25] demonstrated enhanced PSMA expression under ADT in LuCaP patient-derived xenograft mouse models. Taken together, in vitro and in vivo data demonstrate ARB-induced elevation of PSMA expression in various human prostate cancer cell models which may translate into improved RLT radiation delivery.

In line with our in vitro and in vivo data, the patient case supports the translational potential of ARB before ^177^Lu-PSMA RLT. Emmett et al. demonstrated significant reduction in ^68^Ga-PSMA intensity in 86% of men with castrate-sensitive PC as early as day 9 after commencing initial treatment with ADT. In contrast, and in accordance with our findings, all men with mCRPC receiving ARB demonstrated an increase in intensity of ^68^Ga-PSMA PET by day 9 compared with baseline levels [19]. These results might implicate that the effect of ARB on PSMA expression depends on PC stage. While the reduction of PSMA levels, as detected by ^68^Ga-PSMA PET/CT, in hormone-naïve cancer might be due to more effective tumor shrinkage by enzalutamide, ARB in advanced PC might result in PSMA induction with weaker anti-tumor effects. The AR pathway mediates survival in PC; in the case of androgen-independent disease, the initiation of cell death via AR-signaling might be impeded. Together, the effect of ARB on PSMA remains controversial and is an area of active research (e.g., NCT04279561), as the exact role of PSMA in pathogenesis of PC is still poorly understood. Yao et al. showed that the folate hydrolase activity of PSMA may provide a growth advantage in environments with low or physiological folate levels [26]. Other studies reported the involvement of PSMA in cell survival and cell proliferation signaling pathways such as PI3K [27,28] and MAPK [29]. Kaittanis et al. identified a novel oncogenic signaling role for PSMA. PSMA activated the metabotropic glutamate receptor, leading to the PTEN-independent activation of PI3K/AKT signaling; inhibition of PSMA led to tumor shrinkage which was enhanced by concomitant treatment with enzalutamide [28]. These results indicate that PSMA might be involved in establishing the PI3K-AR regulatory loop. However, the complex relationship between PSMA and the androgen axis still needs to be explored further to provide a better understanding of PSMA expression changes under ARB in different PC stages.

### Limitations

Data presented in this study for PSMA-low PC are in line with previous preclinical and clinical findings for PSMA-high tumors. Here, we demonstrate that enzalutamide increases PSMA expression in PSMA-low cancer in vitro, in vivo, and in a clinical case report. However, despite the evidence reported here, the study has a few limitations which need to be addressed.

First, male mice did not undergo additional surgical or chemical castration. It is unknown how androgen depletion may affect PSMA expression in a preclinical setting. Second, baseline to follow-up 22Rv1 tumor size increased significantly under enzalutamide in vivo. PSMA expression after enzalutamide remains significantly increased when tumor size is corrected for partial volume effects; however, tumor growth may have contributed to an overestimation of changes in PSMA expression using PET. Third, it has to be investigated whether ARB-induced increases in PSMA expression will translate into increased radiation delivery and enhanced RLT efficacy in PSMA-low cancer. Several studies demonstrate positive association for ^68^Ga-PSMA and ^177^Lu-PSMA uptake in animal models [30,31]. However, more preclinical data on increased ^177^Lu-PSMA tumor uptake and radiation dose following enzalutamide pretreatment are needed to create solid evidence which in the future might be assessed in clinical trials.

## 4. Materials and Methods

### 4.1. Cell Culture

All cell lines were a kind gift from Dr. Johannes Czernin’s group (University of California Los Angeles). Cells were cultured in Rosewell Park Memorial Institute (RPMI) 1640 medium supplemented with 10% fetal bovine serum and 1% penicillin/streptomycin solution at 37 °C and 5% CO_2_. Cells were routinely assessed for mycoplasma contamination using the Venor^®^GeM OneStep kit (Minerva Biolabs, Berlin, Germany). All used cell lines underwent polymorphic short tandem repeat loci (STRs) profiling to rule out cross-contaminations (Microsynth, Balgach, Switzerland). STR loci were amplified using the PowerPlex^®^ 16 HS System (Promega, Walldrof, Germany). Fragment analysis was done on an ABI3730xl (Life Technologies, Carlsbad, CA, USA) and the resulting data were analyzed with GeneMarker HID software (Softgenetics, State College, PA, USA).

### 4.2. Animals and ARB

All animal studies were performed in accordance with the recommendations of the Society for Laboratory Animal Science (GV-SOLAS) and the European Health Law of the Federation of Laboratory Animal Science Associations (FELASA). The protocol was approved by the North Rhine-Westphalia State Agency for Nature, Environment and Consumer Protection (LANUV), Germany (permit number: AZ.81-02.04.2018.A133). Intact male, 6–10-week-old NOD/Scid gamma mice were housed under pathogen-free conditions. Water and food were provided ad libitum. Mice were injected subcutaneously with 2 × 10^6^ 22Rv1 cells in matrigel/PBS into the shoulder region. Tumor growth was monitored by CT. Animals were sacrificed upon reaching any of predefined termination criteria, including general and social habitus, apathy, ulceration, severe weight loss, tumor size ≥2 cm^3^ or other signs of deteriorating condition. Mice bearing 22Rv1 xenografts (*n* = 9) were treated with 10 mg/kg enzalutamide (Sellekchem) diluted in 68% PEG-200 (Merck, Darmstadt, Germany), 30% Transcutol (Gattefosse, Lyon, France), 1% Labrasol (Gattefosse) and 1% Tween-80 (Sigma Aldrich, Taufkirchen, Germany) 5 times a week for 2 weeks by oral gavage. Treatment with enzalutamide was started as soon as the tumors were palpable (approx. day 14–21 post-inoculation). Average pretreatment tumor volume was mean ± SD: 0.14 ± 0.16 cm^3^ and average weight was mean ± SD: 27.8 ± 2.5 g. ^68^Ga-PSMA PET/CT images of mice bearing C4-2 and LNCaP xenografts were kindly provided Dr. Johannes Czernin’s group (University of California Los Angeles). The design for the in vivo experiments is outlined in Figure 3A.

### 4.3. PET/CT

^68^Ga-PSMA-11 was obtained from ABX. The radiolabeling reaction of ^68^Ga-PSMA-11 was performed by means of Modular-Lab easy using the commercially available reagent kit supplied by Eckert & Ziegler Eurotope GmbH (Berlin, Germany). The radiochemical purity was determined with radio-HPLC and TLC (iTLC-SG) and exceeded 98% for ^68^Ga-PSMA-11. Static PET/CT images of 22Rv1 xenografts were acquired 60 min after intraperitoneal injection of approximately 3 MBq ^68^Ga-PSMA-11 using the β-CUBE (PET) and X-CUBE (CT; MOLECUBES, Gent, Belgium). C4-2 and LNCaP xenografts were injected with 1.1 Mbq ^68^Ga-PSMA-11 and imaged with Genesys8 PET/CT. Mice were imaged in temperature-controlled beds with continuous monitoring of breathing frequency. Image acquisition time was 15 min. Images were reconstructed using an iterative reconstruction algorithm (ISRA, 30 iterations) with attenuation correction of the corresponding CT image. PET data were reconstructed into a 192 × 192 transverse matrix, producing a 400 μm isometric voxel size. PET images were evaluated by analysis of decay-corrected injected activity per gram of tissue (%IA/g). The x-ray source was a tungsten anode (peak voltage, 50 kVp; tube current, 350 μA; 0.8 mm aluminum 759 filter). The detector used was a cesium iodide (CsI) flat-panel, building up a screen with 1536 × 864 pixels. Measurements were carried for individual 120 ms exposures, with angular sampling intervals of 960 exposures per rotation, for a total of 7 rotations and a total exposure time of 6 s. Tumor volume was calculated using PMOD software (version 4.1, PMOD Technologies Ltd., Zürich, Switzerland). The entire tumor volume of interest from CT was copied to the PET dataset to calculate the mean %IA/g. Partial volume effects due to varying tumor sizes were excluded by applying a partial volume correction (Supplementary Figure S2). A partial volume correction factor profile was fitted to an asymptotic curve (Supplementary Figure S2B) with a dependency on the cavity volume measured in a mouse-like phantom (Supplementary Figure S2C). The partial volume correction factor was calculated by dividing the calibrated activity concentration by the mean calibrated activity contained in the PET reconstructed images.

### 4.4. Flow Cytometry

Cells were seeded at a density of 1 × 105/mL (22Rv1), 0.5 × 105/mL (C4-2) or 0.7 × 105/mL (LNCaP) in 6-well plates. Medium was changed every other day. On the day of medium change, cells were treated with 1 µM DMSO (vehicle) or 10 µM enzalutamide. Single-cell suspensions were stained with anti-human PSMA-APC antibody (clone: REA408, Miltenyi Biotec, Bergisch Gladbach, Germany) in PBS according to the manufacturer’s instructions or incubated with 50 µL PBS only (unstained control). Samples were measured after 1, 2 or 3 weeks of treatment with a CytoFlex S flow cytometer (Beckman Coulter, Munich, Germany) and analyzed using FlowJo software (Tri Star Inc., Ashland, OR, USA).

### 4.5. IHC

Cell pellets consisting of ~1 × 10^6^ cells were embedded in paraffin. 3 µm sections from formalin-fixed paraffin-embedded (FFPE) blocks were cut and dewaxed. Samples were pretreated with tris-based buffer (CC1, Ventana medical systems, Hoffmann-La Roche, Basel, Switzerland) at 90 °C for 32 min for PSMA and 64 min for AR, respectively. All reactions were performed on an automated staining device (Ventana Benchmark, Tucson, AZ, USA). Samples were stained for PSMA (clone 3E6, Dako, 1:50) and androgen receptor (AR; clone SP107, Cell Marque, 1:2000) and detected by OptiView DAB IHC Detection Kit (Ventana Medical Systems Inc., Tucson, AZ, USA) Percentage of stained cells was semi-quantitatively analyzed on an Olympus BX 50 device (Olympus, Tokio, Japan) by a genitourinary pathologist. For AR-IHC, only nuclear immunoreactivity and for PSMA-IHC, only cytoplasmic immunoreactivity was evaluated. Control tissue microarray for IHC stainings can be found in Supplementary Figure S3.

### 4.6. Statistics

All data were analyzed with GraphPad Prism software (version 9.0.1, GraphPad software Inc., CA, USA). Data are presented as mean ± standard deviation (SD). Data were assessed for normal Gaussian distribution and parametric or non-parametric tests were used accordingly. Statistical significance between two unpaired groups was assessed using the unpaired t-test with Welch’s correction for unequal SD. Statistical significance between two paired groups was assessed using the Wilcoxon matched-pairs signed-rank test. Statistical significance between more than two unpaired groups was assessed using the ordinary one-way ANOVA test with Sidak’s multiple comparisons test. *P*-values below 0.05 were considered statistically significant. Statistically significant data are indicated by asterisks (* *p* < 0.05, ** *p* < 0.01, *** *p* < 0.001, **** *p* < 0.0001).

## 5. Conclusions

In conclusion, enzalutamide induces PSMA in cell culture, a xenograft model, and in a patient case of mCRPC. Pre-treatment with ARB might thus render patients with PSMA-low PC eligible for RLT. Although the mechanisms underlying lack of responsiveness to RLT are very complex and still under investigation, PSMA expression has been identified as a strong predictor of RLT response and long survival [9,17]. Therefore, the impact of enzalutamide on PSMA expression as well as RLT eligibility and efficacy should further be assessed in prospective clinical trials.

## Data Availability

The data that support the findings of this study are available from the corresponding author upon reasonable request.

## Supplementary Materials

The following are available online at https://www.mdpi.com/article/10.3390/ijms22147431/s1, Figure S1: ^68^Ga-PSMA PET/CT before and after enzalutamide.; Figure S2: Empiric partial volume effect (PVE) correction data and methodology. Figure S3: Control tissue microarray for immunohistochemistry stainings (PSMA).
