# Supplementary material for: Enzalutamide Enhances PSMA Expression of PSMA-Low Prostate Cancer
Source: Int J Mol Sci. 2021 Jul 11;22(14):7431. doi: 10.3390/ijms22147431 (PMC8304389; doi:10.3390/ijms22147431)
Supplement: Supplementary file 1 [file ijms-22-07431-s001.zip › ijms-1248615-supplementary.pdf]

Enzalutamide enhances PSMA expression of PSMA-low prostate cancer – Supplemental Figures

Magdalena Staniszewska 1, Pedro Fragoso Costa 1, Matthias Eiber 2, Jasmin M. Klose 1, Jasmin Wosniack 1, Hen-ning Reis 3, Tibor Szarvas 4,5, Boris Hadaschik 4, Katharina Lückerath 1,6, Ken Herrmann 1, Wolfgang P. Fendler 1,* and Janette Iking 1,*#

1 Department of Nuclear Medicine, University of Duisburg-Essen and German Cancer Consortium (DKTK)-University Hospital Essen, Hufelandstraße 55, D-45147 Essen, Germany;
2 Department of Nuclear Medicine, Klinikum rechts der Isar, Technical University of Munich, Ismaninger Straße 22, D-81675 Munich, Germany;
3 Institute of Pathology, University of Duisburg-Essen, University Hospital Essen, Hufelandstraße 55, D-45147 Essen, Germany;
4 Department of Urology, University of Duisburg-Essen and German Cancer Consortium (DKTK)-University Hospital Essen, Hufelandstraße 55, D-45147 Essen, Germany;
5 Department of Urology, Semmelweis University, Ulloi ut 78/b, 1082, Budapest, Hungary
6 Department of Molecular and Medical Pharmacology, David Geffen School of Medicine, University of Califor-nia Los Angeles, 650 Charles E young Drive S, Los Angeles, CA 90095, USA;
*** The authors contributed equally to this study.**
# Correspondence: janette.iking@uk-essen.de

Universitätsmedizin Essen
Universitätsklinikum
Klinik für Nuklearmedizin

Suppl. Fig. 1 ^{68}Ga-PSMA PET/CT before and after enzalutamide.

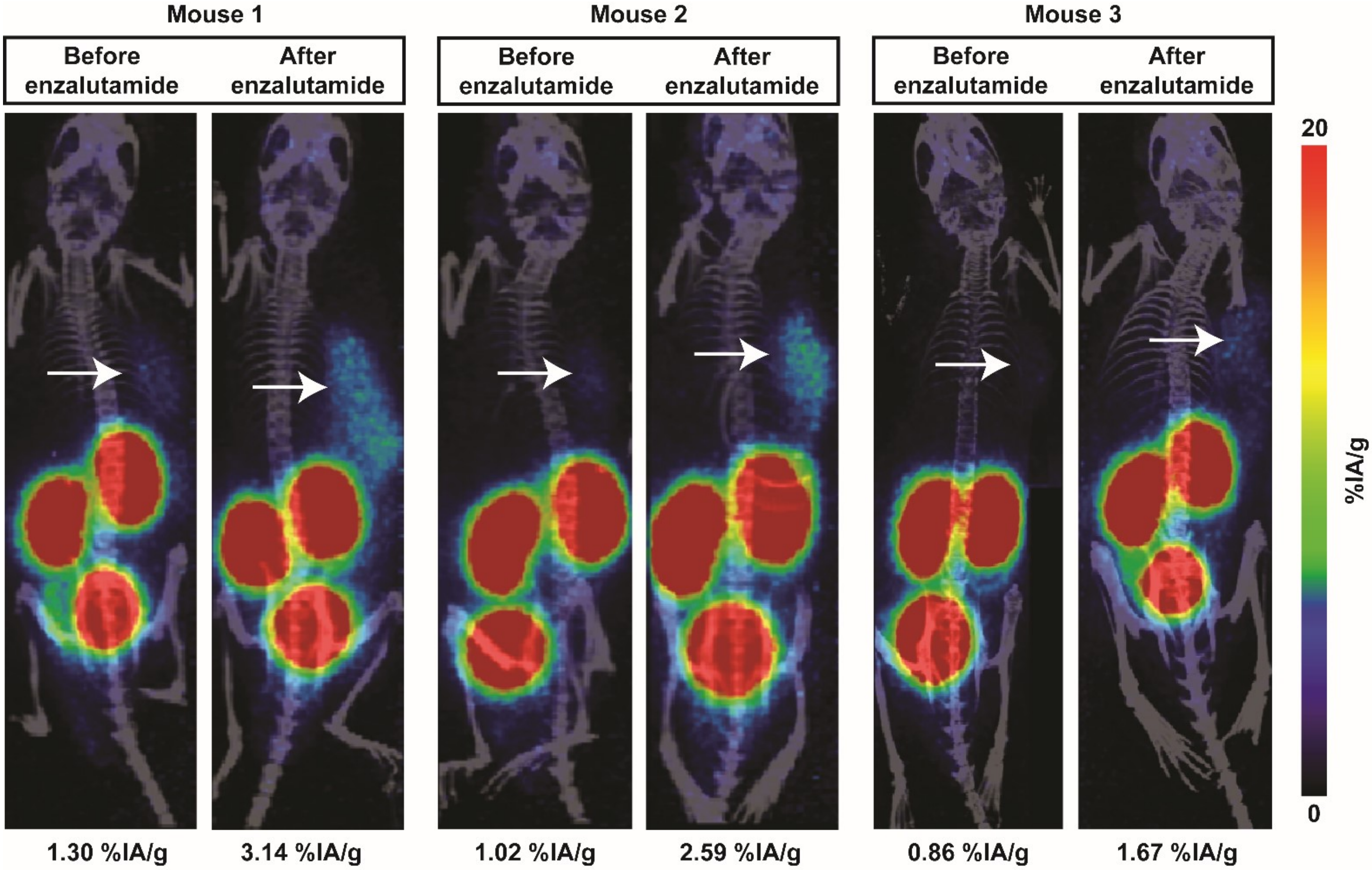

Fig S1. ^{68}Ga-PSMA PET/CT before and after enzalutamide. Representative maximum intensity projections (MIP) of three different mice bearing 22Rv1 tumors (arrow) before (baseline) and after a two-week treatment with enzalutamide are shown (n=9). Average ^{68}Ga-PSMA tumor uptake is given below the image. **Abbreviations**: %IA/g, % injected activity per gram tissue.

Suppl. Fig. 2 Empiric partial volume effect (PVE) correction data and methodology.

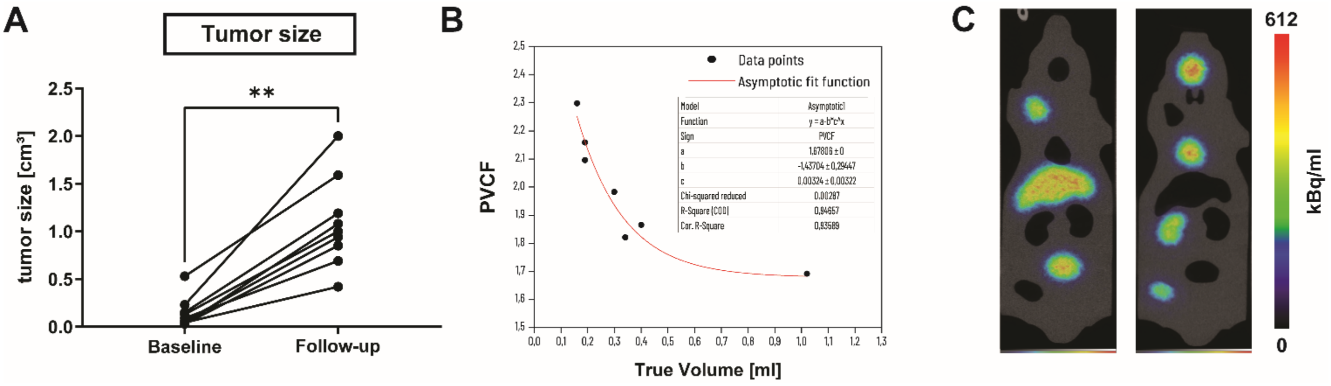

Fig S2. Empiric partial volume effect (PVE) correction data and methodology. Since the tumor size at baseline is significantly lower than at follow-up (A), it is appropriate to correct for partial volume effects (PVE). A partial volume correction factor (PVCF) profile was fitted to an asymptotic curve **(B)** with a dependency on the cavity volume measured in a mouse-like phantom **(C)**. The PVCF was calculated by dividing the calibrated activity concentration by the mean calibrated activity contained in the PET reconstructed images. **Statistics**: Wilcoxon matched-pairs signed rank test. **p≤0.01.

Suppl. Fig 3 Control tissue microarray for immunohistochemistry stainings.

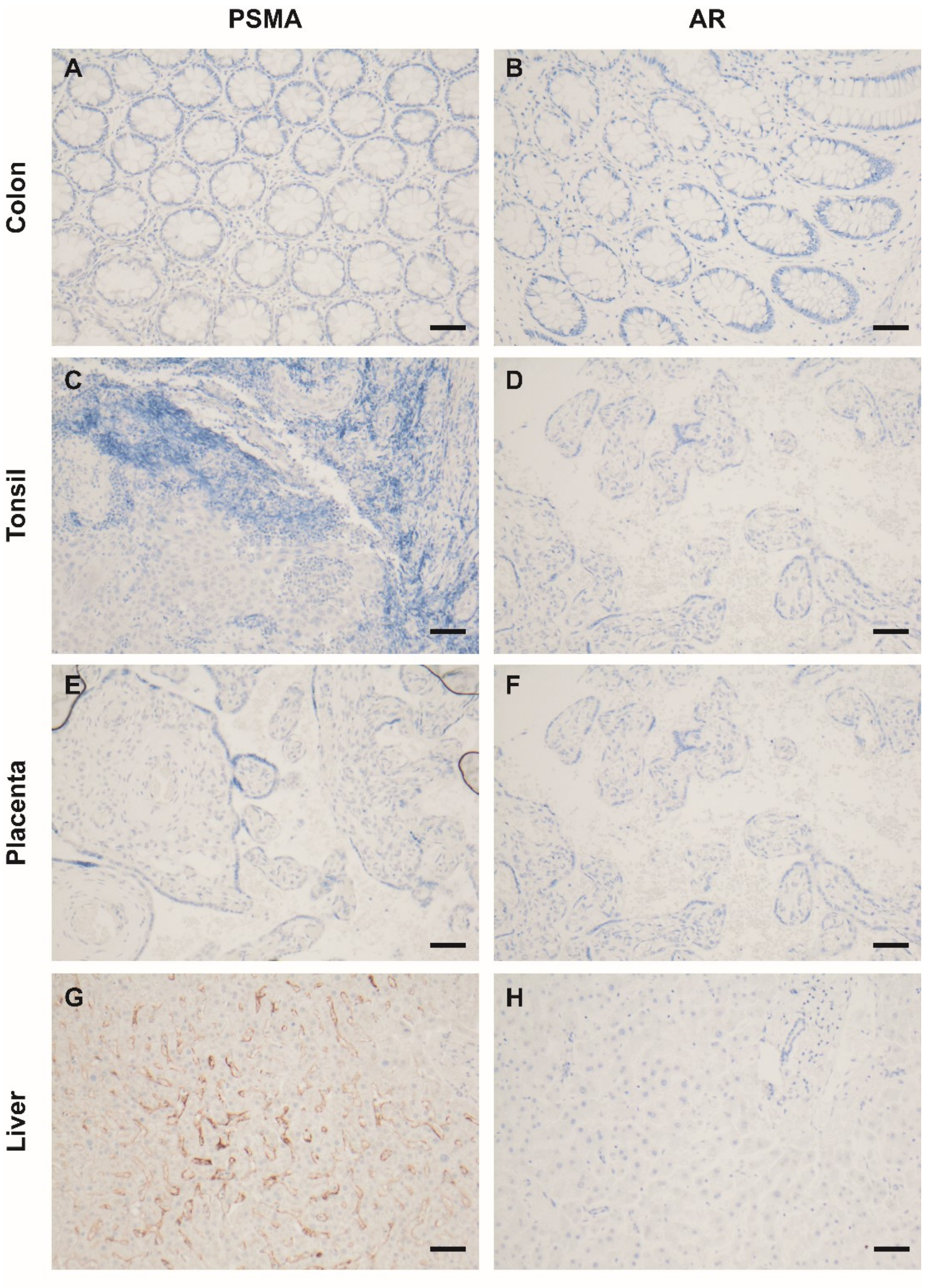

Fig S3. Control tissue microarray for immunohistochemistry stainings. Colon, tonsil and placenta serve as negative controls for PSMA and AR. Live tissue is negative for AR and typically positive for PSMA. **Scale:** 50 µm.

*
